# Identifying pre-outbreak signals of hand, foot and mouth disease based on landscape dynamic network marker

**DOI:** 10.1186/s12879-020-05709-w

**Published:** 2021-01-15

**Authors:** Xuhang Zhang, Rong Xie, Zhengrong Liu, Yucong Pan, Rui Liu, Pei Chen

**Affiliations:** 1 School of Computer Science and Engineering, South China University of Technology, Guangzhou, 510006 China; 2 School of Information, Guangdong University of Finance and Economics, Guangzhou, 510320 China; 3 School of Mathematics, South China University of Technology, Guangzhou, 510640 China; 4 Guangdong Science and Technology Infrastructure Center, Guangzhou, 510033 China

**Keywords:** Hand, foot and mouth disease (HFMD) outbreaks, Pre-outbreak signals, Critical transition, City network, Landscape dynamic network marker (L-DNM)

## Abstract

**Background:**

The high incidence, seasonal pattern and frequent outbreaks of hand, foot and mouth disease (HFMD) represent a threat for billions of children around the world. Detecting pre-outbreak signals of HFMD facilitates the timely implementation of appropriate control measures. However, real-time prediction of HFMD outbreaks is usually challenging because of its complexity intertwining both biological systems and social systems.

**Results:**

By mining the dynamical information from city networks and horizontal high-dimensional data, we developed the landscape dynamic network marker (L-DNM) method to detect pre-outbreak signals prior to the catastrophic transition into HFMD outbreaks. In addition, we set up multi-level early warnings to achieve the purpose of distinguishing the outbreak scale. Specifically, we collected the historical information of clinic visits caused by HFMD infection between years 2009 and 2018 respectively from public records of Tokyo, Hokkaido, and Osaka, Japan. When applied to the city networks we modelled, our method successfully identified pre-outbreak signals in an average 5 weeks ahead of the HFMD outbreak. Moreover, from the performance comparisons with other methods, it is seen that the L-DNM based system performs better when given only the records of clinic visits.

**Conclusions:**

The study on the dynamical changes of clinic visits in local district networks reveals the dynamic or landscapes of HFMD spread at the network level. Moreover, the results of this study can be used as quantitative references for disease control during the HFMD outbreak seasons.

**Supplementary Information:**

The online version contains supplementary material available at 10.1186/s12879-020-05709-w.

## Background

Hand, foot and mouth disease (HFMD) is a global infectious disease that has been reported in many countries around the world, especially in the Asia-Pacific region. Since June 2019, a severe outbreak of HFMD has occurred in multiple regions of Japan, which attracted people’s attention once again. Generally, the main etiologic agents of HFMD are human enterovirus 71 (EV-A71) and Coxsackievirus 16 (CV-A16) [1]. Although usually mild—with symptoms limited to 38 °C fever, malaise, rashes on the volar regions of the hands and feet, herpangina and difficulty in eating and drinking, infection may lead to severe complications of the nervous or cardiopulmonary systems [2]. For some cases, HFMD results in long-term sequelae such as cognitive and motor disorders [3, 4] or even death. Moreover, global epidemiology of HFMD and its social consequences have been documented in the past decade, especially in Japan [5], Singapore [6] and mainland China [7, 8], where large-scale outbreaks of HFMD have occurred, resulting in a substantial costs of epidemics to the economy and global public health concerns.

Early recognition of pre-outbreak signals of HFMD and timely preventive measures can greatly reduce the magnitude and distribution of infection. However, due to the lack of public health infrastructure and economic incentives, which lead to the inability to recognize the potential progression of an epidemic [9, 10], it is still challenging to predict the HFMD outbreaks in a timely manner. Fortunately, with the disclosure of real-time monitoring data, an appropriate method of calculation is needed to identify pre-outbreak signals based on available data of HFMD, thus simplifying the process of data collection and monitoring.

**Fig. 1:**
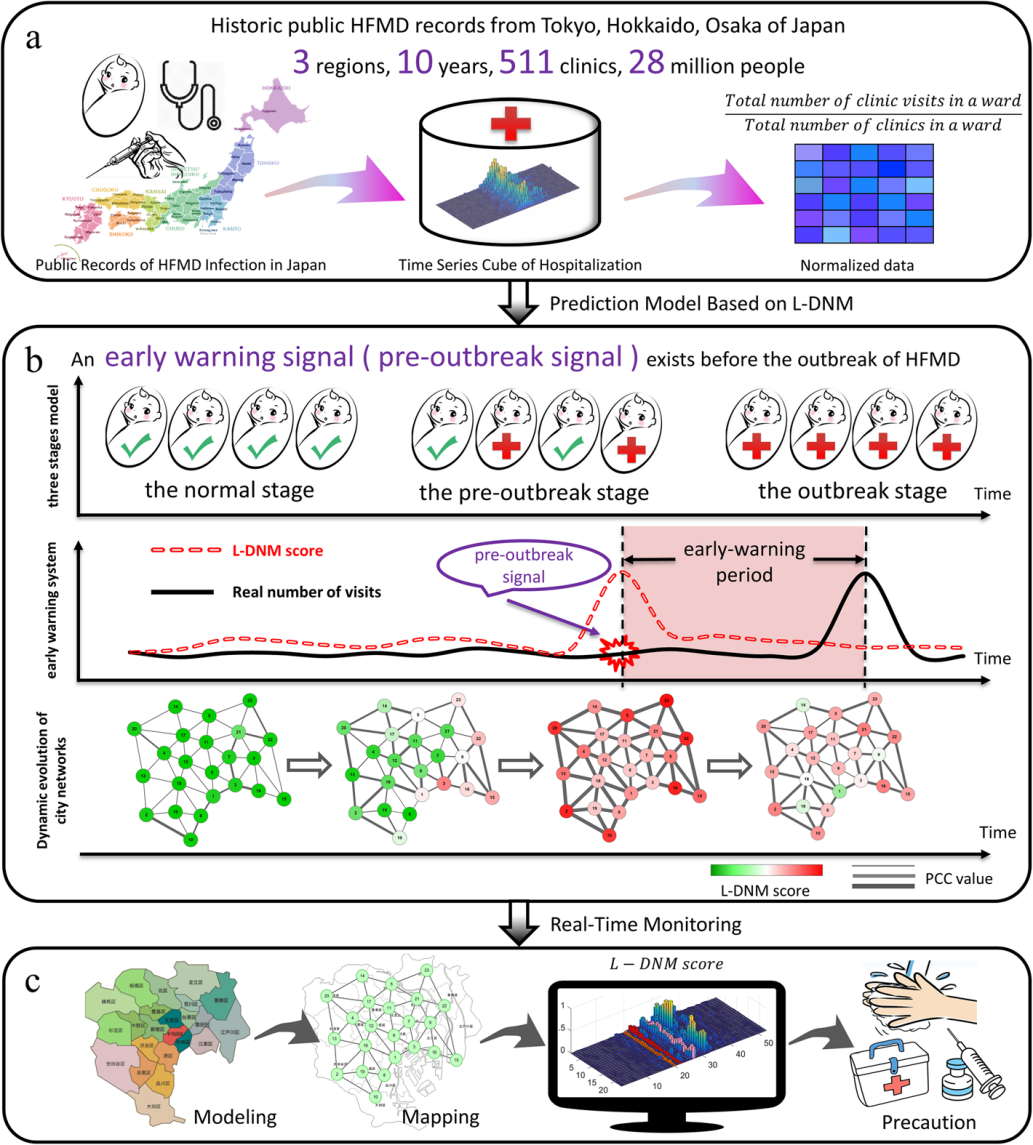
Schematic diagram to detect pre-outbreak signals of HFMD based on the L-DNM method. **a** The historical information of clinics hospitalization due to HFMD infection from January 1, 2009 to December 31, 2018, was collected from public records in Tokyo, Hokkaido and Osaka, Japan. **b** According to the DNM theory, the process of HFMD outbreaks is divided into three stages, including the normal stage, the pre-outbreak stage and the outbreak stage. The sudden increase in the DNM score indicates a transition from the normal stage to the pre-outbreak stage, i.e., the critical point before the upcoming outbreak of HFMD that results in an increase in clinical visits. **c** Based on the historical and current clinical records and geographic characteristics of a region, the DNM score provides an early warning signal of an upcoming outbreak of HFMD as a real-time indicator

In this study, we develop a computational method, the so-called landscape dynamic network marker (L-DNM), to detect the early-warning signals of HFMD outbreaks. First, in a dynamical modelling way, the stage before the outbreak of HFMD is regarded as a pre-transition stage, immediately after which the system undergoes a critical transition. Then the dynamical process of an epidemic system can be roughly modelled as three stages, i.e., a normal stage, a pre-outbreak stage and an outbreak stage. According to L-DNM method, when the system transits from the normal stage to the pre-outbreak stage, the city network changes significantly and L-DNM score rises sharply (Fig. 1b). Unlike the traditional detection of the outbreak stage, the L-DNM method can identify the pre-outbreak stage that generally has no clear abnormalities but with high potential of state transition into a severe and irreversible stage. The proposed L-DNM is mainly based on a theoretical background of dynamic network biomarker (DNB) method [11], which identifies the critical state of complex diseases by analyzing the dynamics of driven biomolecules (i.e., a group of genes and proteins that are the leading factors to the critical state transition). The DNB method has been applied to a number of biological progresses and achieved satisfactory results, including identifying the critical points of cell fate decision [12] and cellular differentiation [13], and detecting the critical period during various biological processes [14–17]. Different from micro-biomolecular networks which are constructed mainly based on regulations among genes and proteins, macro-city networks can be built according to the geographical distribution and population mobility among regions [18]. Based on such city network, the L-DNM approach helps to study the dynamics of epidemic and effectively detect the early-warning signals of any potential disease outbreaks. We applied the L-DNM method to a set of real-time clinic hospitalization records of HFMD, which were collected from 175 clinics distributed in 23 wards of Tokyo, Japan, 139 clinics distributed in 30 wards of Hokkaido, Japan, and 197 clinics distributed in 11 wards of Osaka, Japan (Fig. 1a). The results show that L-DNM method effectively monitors the epidemic process of HFMD and successfully detect the pre-outbreak signals about 5 weeks before the actual peak of the outpatient number. Besides, for each outbreak, the L-DNM method reveals the temporal and spatial information of HFMD transmission at the city network level. Therefore, such method is of great applicable potential in public health management, which may help to develop new control strategies for HFMD before its outbreaks (Fig. 1c).

## Methods

### Theoretical background

The theoretical basis of this research is the dynamic network biomarker (DNB) method [11]. Specifically, when a complex dynamic system approaches a critical point, there exists a dominant group defined as the DNB group that satisfies the following three properties [19]:

i) The standard deviation (*SD*_*in*_) for any member in the DNB group increases sharply;
ii) The correlation (*PCC*_*in*_) between any pair of members in the DNB group increases rapidly;
iii) The correlation (*PCC*_*out*_) between one member of the DNB group and any other non-DNB member decreases rapidly.

Based on the above three properties, it is possible to find a group defined as the dynamical network marker (DNM) group of highly correlated variables with strong fluctuations, the emergence of which means an upcoming state transition during a biological process. Then, these three properties are applied to detect the critical state as an early warning signal of diseases. In order to quantify the critical state, *I*_*DNM*_ is used as a composite index to quantitatively measure the critical signal:

$$ {I}_{DNM}=\frac{PCC_{in}}{PCC_{out}}{SD}_{in}. $$

Whenever the *I*_*DNM*_ score increases significantly, it is considered that the system is close to the critical transition point. The detailed description and derivation of DNB can be found in the reference [20] and its Supplemental Information.

Based on the DNB theory, the dynamical process of HFMD outbreaks is roughly divided into the following three stages (Fig. 1b) similar to the dynamics of disease progression [11]: the normal stage, which has stable dynamic characteristics with high resilience; the pre-outbreak stage, which is dynamically unstable and with low resilience. In this stage, the epidemic is still controllable through appropriate measures; and the outbreak stage, which is another stable stage with high resilience. Obviously, identifying the warning signals in the pre-outbreak stage is the key to implement effective control management to prevent HFMD outbreaks. However, unlike the outbreak stage with a large number of clinic visits, there is little significant difference between the pre-outbreak stage and the normal stage. In order to detect the pre-outbreak signals of HFMD more accurately, we developed the landscape dynamic network marker (L-DNM) method which is applied to the historical records of HFMD and analyze the local and global city network.

### Landscape dynamic network marker (L-DNM)

**Fig. 2:**
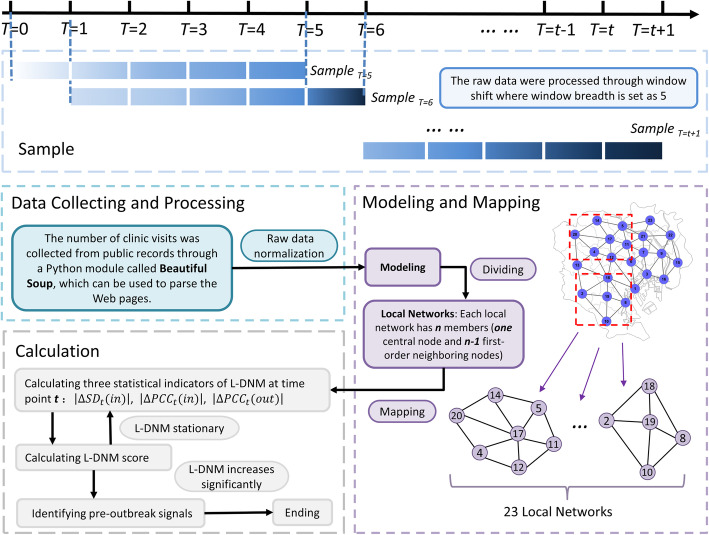
The algorithm of landscape dynamic network marker. The flow chart above shows how the algorithm works based on city networks and the historical information of clinic hospitalization. Regarding a point *T* = *t* (*t* > 5) as a candidate tipping point, L-DNM scores can be calculated. If the L-DNM score increases significantly, the candidate *T* = *t* is determined as the identified tipping point, and the algorithm ends. Otherwise, if there is no significant change in the score, then *T* = *t* is classified as a time point belonging to the normal stage, and the algorithm continues with *T* = *t* + 1 being a new candidate tipping point

The L-DNM method is illustrated in Fig. 2 and described in the following three steps.

#### Modeling and mapping

**Fig. 3:**
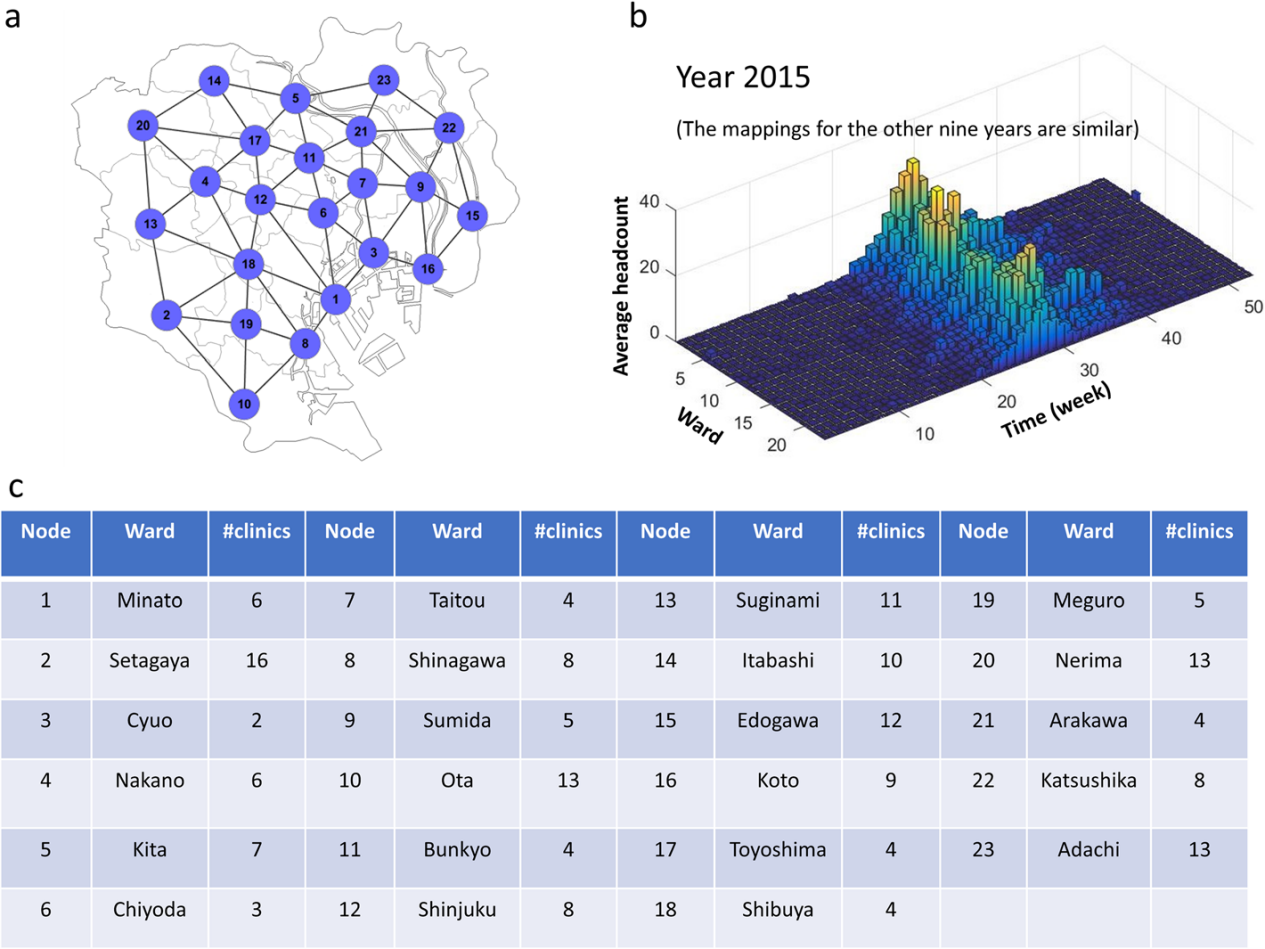
The city network model of Tokyo. **a** A 23-node network model was constructed based on the geographic information and adjacent relationships of the 23 wards. **b** For each week, the average number of clinic visits in the ward was mapped to the corresponding node, through which we obtained a data matrix of 23 rows/wards and 522 columns/weeks. **c** A detailed list of correspondences between wards and nodes, including the number of clinics in each ward

In the first step, we construct the city network based on the geographic distribution of the wards/districts and their adjacent information. In the network, each node represents a ward, while each edge represents the adjacent relation between two wards. Then the records of HFMD outpatients within a 5-week sliding window are mapped to the city network. The city network model is demonstrated as in Fig. 3.

#### Calculating L-DNM score

In the second step, the city network is divided into local networks piece by piece, each of which contain a central node/ward and all of its first-order neighbors. For a local network with *n* members (i.e., a central node and *n*-1 first-order neighbors), we calculate the local-network index *I*_*t*_ at a sampling point *t* by the following definition:

$$ {I}_t=\left|\Delta {SD}_t(in)\right|\left[\left|\Delta {PCC}_t(in)\right|+\left|\Delta {PCC}_t(out)\right|\right], $$

where

$$ \left|\Delta {SD}_t(in)\right|=\frac{\sum_{i=1}^n\left|{SD}_t(i)-{SD}_{t-1}(i)\right|}{n} $$

is the average differential standard deviation (in absolute value) of the nodes in the local network;

$$ \left|\Delta {PCC}_t(in)\right|=\frac{\sum_{i=1,j=1}^n\left|{PCC}_t\left(i,j\right)-{PCC}_{t-1}\left(i,j\right)\right|}{n\times n} $$

is the average differential Pearson’s correlation coefficient (in absolute value) within the local network. That is, nodes *i* and *j* are both in the same local network;

$$ \left|\Delta {PCC}_t(out)\right|=\frac{\sum_{i=1,j=1}^n\left|{PCC}_t\left(i,j\right)-{PCC}_{t-1}\left(i,j\right)\right|}{n\times n} $$

is the average differential Pearson’s correlation coefficient (in absolute value) between a member (node *i*) in the local network and that (node *j*) outside.

Clearly, during the pre-outbreak stage, i.e., at a sampling point *t* ∈critical state, there are three cases for each local network:

- In the local network, all the nodes are DNM members;
- In the local network, there are DNM and non-DNM members;
- In the local network, all the nodes are non-DNM members.

As shown in Table 1, for the above three cases respectively, there are significant changes for the statistical indices of each local network. Obviously, each node or ward corresponds to an index value, *I*_*t*_, which can quantitatively characterizes the criticality of each node at a sampling point *t*. As time evolves, a landscape can be constructed based on those *I*_*t*_ scores of all nodes. According to Table 1, the *I*_*t*_ score of each DNM node increases sharply based on the three statistical conditions of DNM. Therefore, during the process of transition from the normal stage to the outbreak stage, the DNM group helps to detect the early warning signal of the critical state.

**Table 1:** Critical behaviours of a central node’s L-DNM score for different cases

Case	Nodes		***SD***_***t***_	∣Δ***SD***_***t***_(***in***)∣	***PCC***_***t***_(***in***)	|Δ***PCC***_***t***_(***in***)|	***PCC***_***t***_(***out***)	|Δ***PCC***_***t***_(***out***)|	***I***_***t***_
1	All DNM		↗	↗	↗	↗	D↗	↗	↗
							N↘		
2	DNM and non-DNM	D	↗	↗	D↗	↗	D↗	↗	↗
					N↘	↗	N↘	↗	
		N	→	0	D↘	↗	D↘	↗	
					N→	0	N→	0	
3	All non-DNM		→	0	→	0	D↘	↗	0
							N→	0	

Notes: When the system moves from time point *t* − 1 to *t*, it is approaching the critical point

1. “↗” represents the increase of variables; “↘” represents the decrease of variables; “→” represents that there is no significant change in the variables;

2. “D” represents the DNM members; “N” represents the non-DNM members;

3. *SD*_*t*_ is the average standard deviation at time point *t*; *PCC*_*t*_(*in*) is the average Pearson’s correlation coefficient between two nodes within the local network; *PCC*_*t*_(*out*) is the average Pearson’s correlation coefficient between a node inside the local network and a node outside

#### Identifying multi-level early warnings

It is observed that a severe outbreak of HMFD occurs in every 2 to 3 years. For example, in 2013, 2015 and 2017, the number of infected patients was significantly more than that in other years. Taking this fact into consideration, we set up a multilevel early warning system, including the mild (orange) warning and the severe (red) warning. Specifically, an adjustable threshold *M*_*threshold*_ is applied to identify the significant changes of *I*_*t*_ scores, which is given as the following formula:

$$ {M}_{threshold}=\frac{\sum_{i=1}^n{I}_t(i)\times \left(t-1\right)}{\sum_{j=1}^{t-1}{\sum}_{i=1}^n{I}_j(i)} $$

where *I*_*t*_(*i*) represents the score *I*_*t*_ of the central node *i* at a time point *t* for the local network with *n* members.

The above threshold is then determined by the specific historical records of a region. For example, based on the clinic hospitalization records of HFMD in Tokyo, a 5-fold-change threshold is considered as the orange warning and an 8-fold-change threshold is regarded as the red warning. In this study, the similar way to determine the adjustable threshold is also applied to the datasets of Hokkaido and Osaka.

### Data processing

#### Data normalization

The process of data normalization is important for predicting outcomes since the population of each ward/district is roughly proportional to the number of clinics. The raw data was averaged by the total number of clinics inside the ward/district.

#### Sliding window

In the calculation process, we processed the original data by window shifting, where the window width was set as 5. In other words, the standard deviation and correlation coefficient were calculated based on the data within every 5 weeks.

## Results

### Identifying pre-outbreak signals of HFMD in Tokyo

The transmission of HFMD is a complicated dynamical system with a lot of biomedical and social factors. Due to the massive number of influencing factors, it is difficult to mathematically describe such transmission dynamics in a high-dimensional space. The sharp or qualitative transition from the normal state to the outbreak state of the local network corresponds to the bifurcation point in the theory of dynamic systems [21]. Based on this theory, if the system approaches the bifurcation point, it will eventually be constrained to one-dimensional or two-dimensional space (i.e., the centre manifold in general sense), where the dynamic system can be expressed in a very simple form. This is the theoretical basis for developing a generic indicator that can detect pre-outbreak signals of HFMD based on observed data.

As shown in Fig. 1, we collected the historical information of clinic hospitalization caused by HFMD infection from January 1, 2009 to December 31, 2018 in Tokyo, Japan. The outbreak point of HFMD was defined as the peak of the hospitalization counts every year. According to the first step of L-DNM method, a 23-node network was constructed based on the geographic distribution of 23 wards and their adjacent relationships (Fig. 3).

**Fig. 4:**
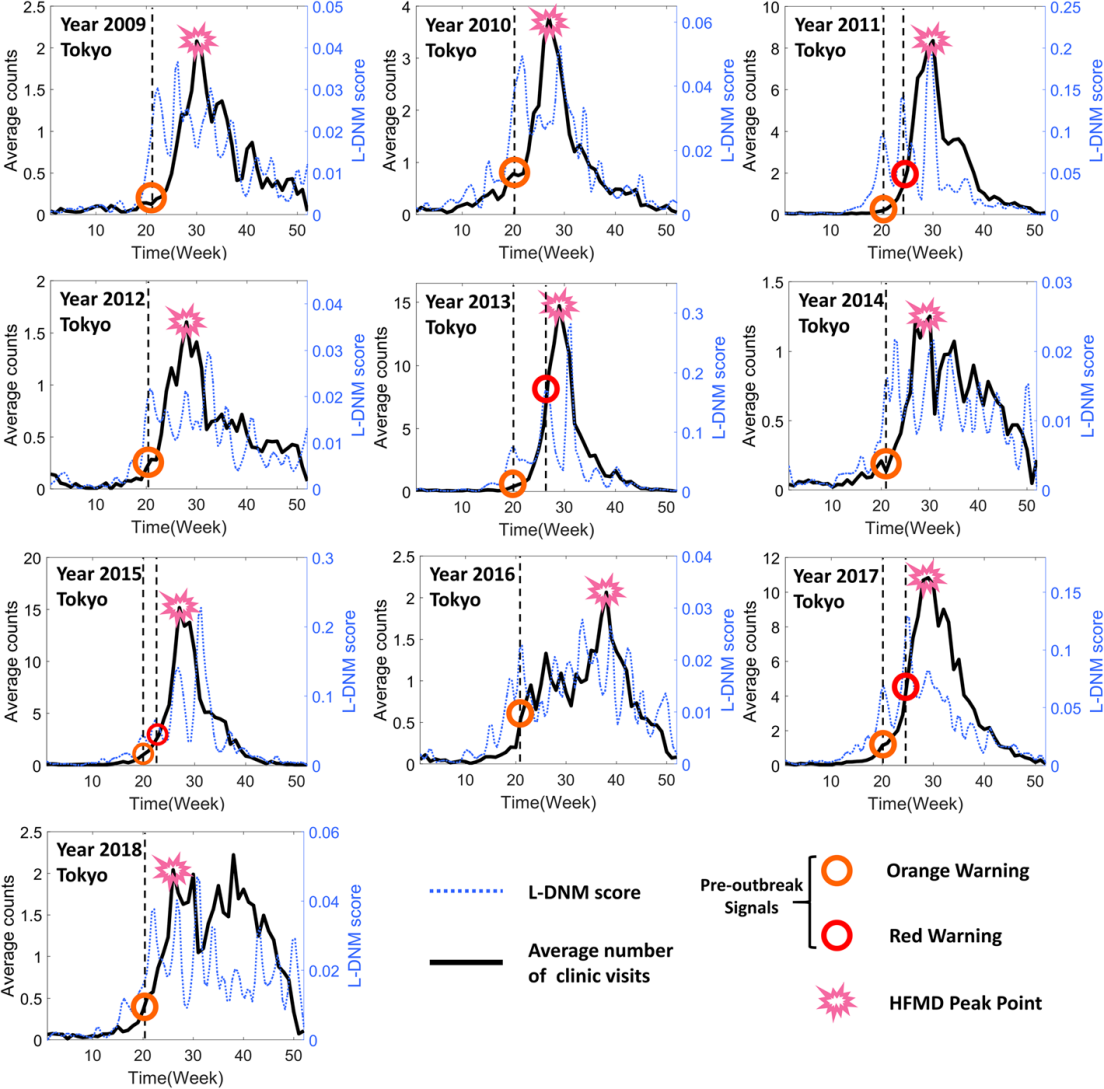
Forecast of seasonal HFMD outbreaks in Tokyo between the years 2009 and 2018. In each subgraph, the left y-axis is the average number of patients in each clinic and the right y-axis is the L-DNM score that was calculated based on a 5-week sliding window scheme; the x-axis represents the period from first week of the year to last week of the year. Besides, the markers of different colors and shapes are used to identify warning signals or outbreak points. Clearly, when the actual number of clinic visits has not increased significantly, significant changes in L-DNM scores have been detected, indicating the presence of pre-outbreak signals

Provided as in Fig. 4, the pre-outbreak signals were identified through L-DNM method for each seasonal outbreak of HFDM. It can be seen that an uncontrollable outbreak of HFMD occurs every 2 to 3 years. For example, in 2011, 2013, 2015 and 2017, the peak of the total hospitalization counts was about four times that of other years. In particular, CV-A6 emerged as a primary causative agent in 2011, causing the largest HFMD epidemic in Japan since 1981 [22]. Since then, CV-A6 has caused large HFMD epidemics every 2 years. In addition, as shown in Fig. 4, the orange warning signal indicates that the infection of HFMD has entered a pre-outbreak stage, and the red signal successfully warns a large outbreak of HFMD. Therefore, for each HFMD outbreak later developing into a large outbreak, the L-DNM score is sensitive and significantly increases about 5 weeks before the actual number of hospitalizations skyrockets.

**Fig. 5:**
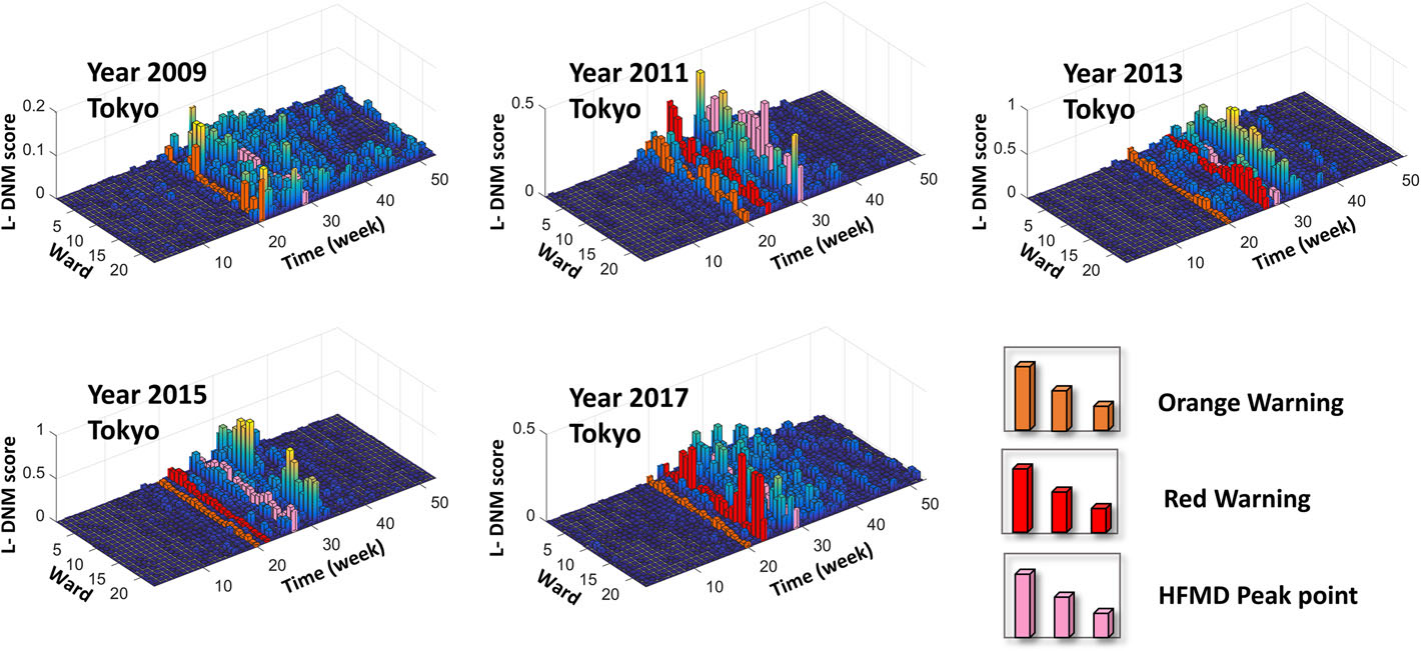
The landscape of L-DNM score for 23 wards in Tokyo. In each landscape figure, the L-DNM scores of 23 wards in Tokyo are presented annually. The orange column points to the first appearance of warning signals, the red column indicates that the scale of infection is expanding and the pink column indicates an outbreak. Obviously, the warning signals are sensitive and effective. For the 10-year landscape figures, please see Figure S1 in additional file [see Additional file 1]

To better illustrate the L-DNM method’s principle, we show the landscape for L-DNM scores of every local network as in Fig. 5. As time evolves, the landscape can be constructed based on the L-DNM scores of all nodes. It can be seen that the first discovered pre-outbreak signal is 4–8 weeks ahead of the HFMD outbreak point defined at the peak of hospitalization counts. The successful prediction of each HFMD outbreak in different regions demonstrates the robustness and effectiveness of L-DNM method in identifying real-time warning signals for infectious diseases.

**Fig. 6:**
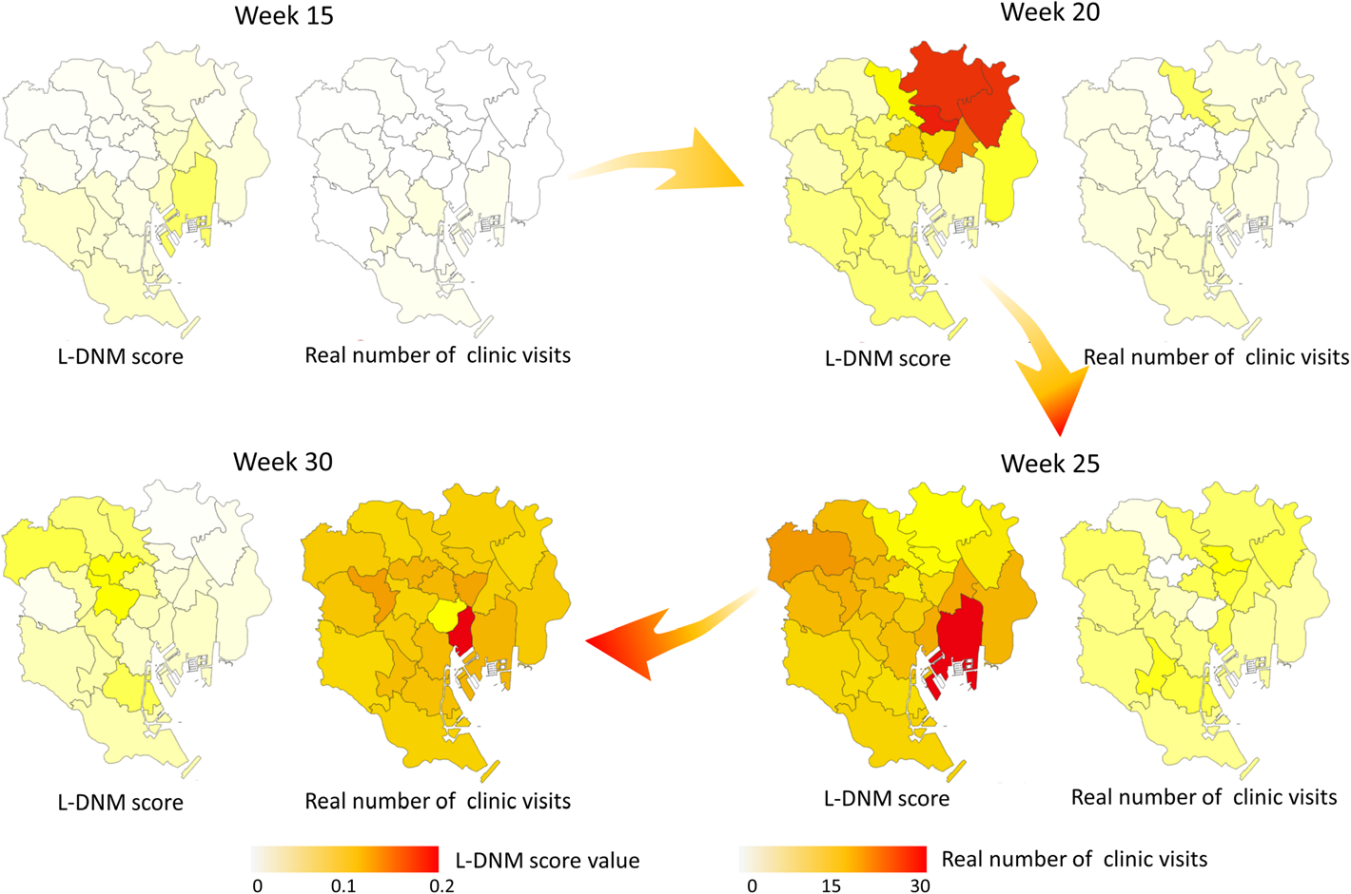
Dynamic evolution map of L-DNM scores in Tokyo. Maps respectively in the 15th week (the normal stage), the 20th week (the pre-outbreak stage), the 25th week (the pre-outbreak stage) and the 30th week (the outbreak stage), are colored by the scaled value of L-DNM score (left of each submap) and the number of real clinic visits (right of each submap). It can be seen that the maps have no significant changes during the normal stage (e.g., the 15th week). However, as the system approaches the pre-outbreak stage (e.g., the 20th–25th week), the maps change dramatically, reflecting the apparent early warning signals of the upcoming outbreak

In addition, we also introduce the dynamic evolution of the transmission network of HFMD in Tokyo. Figure 6 shows that the L-DNM score of each node is mapped to the actual map. When the actual number of clinic visits does not increase significantly as shown, L-DNM method has identified the pre-outbreak signal. In other words, as the system approaches the bifurcation point, the correlation both the local network and adjacent wards increases dramatically, which indicates abnormal changes of the system. The dynamic evolution of city networks reveals the transmission situation and trend of HFDM, and better presents the transmission dynamics at the network level of the system.

### Application of L-DNM in Hokkaido and Osaka

In order to verify the effectiveness of our model, we also applied L-DNM to detect pre-outbreak signals of HFMD in Hokkaido and Osaka, Japan. The results are shown in Figures S2-S6 of Additional File [see Additional file 1].

As can be seen from Figure S2, 30 wards of Hokkaido were modelled as a 30-node city network. Figure S3 shows that there were seven seasonal normal outbreaks and three large-scale outbreaks of HFMD in Hokkaido between year 2009 and year 2018, among which L-DNM method provided pre-outbreak signals to nine outbreaks. It can be seen that the dynamic evolution of L-DNM scores for each local network from Figure S4. The modelling and mapping process of Osaka is similar, which is shown in Figure S5. For Osaka (Figure S6), seven HFMD outbreaks occurred from year 2012 to 2018, among which L-DNM method provided pre-outbreak signals for five outbreaks accurately. After the difference analysis of city networks, the reason we get is that the number of Osaka’s network nodes is relatively small, which leads to the inadequate expression of network information.

### Performance comparison with other methods

**Fig. 7:**
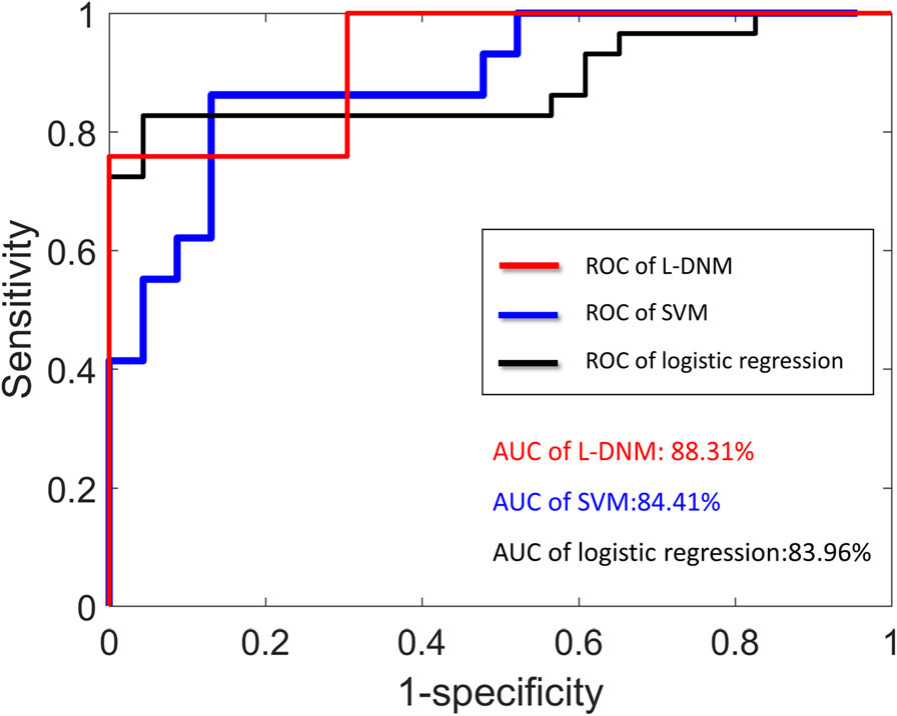
The performance comparisons of L-DNM-based and machine-learning-based methods. It can be seen that the L-DNM-based surveillance system performs better than the logistic regression or SVM using only the records of Tokyo clinic visits. The AUC of L-DNM is 0.8831, while that of support vector machine is 0.8441 and that of logistic regression is 0.8396

According to Tokyo Metropolitan Infectious Disease Surveillance Center, the benchmark for alerting infectious disease epidemics is an average of five patients per clinic. Obviously, the alarm appears too late to take effective preventive measures. In addition, there are no alarm signals in some years, such as 2010, etc. Therefore, if L-DNM method is used, the above shortcomings can be overcome. Moreover, the performance of comparing L-DNM method with machine learning algorithm is shown in Fig. 7. Specifically, logistic regression algorithm and support vector machine (SVM) are applied to infectious disease surveillance system [23–26]. As can be clearly seen from Fig. 7, a DNM-based system performs better than a system based on logistic regression or SVM when only hospitalization records are given. In recent years, deep learning approaches, such as a time series model with long short-term memory (LSTM) [27], have been applied to simulate the seasonality and trends of infectious diseases incidence. But It is necessary for LSTM to collect time-series data for many years, which is unrealistic for some developing countries. And since the sample length and time periods adopted to construct the models might have an impact on the forecasting power, additional data categories, such as meteorological data and search engine query data, are provided to test the robustness of the developed models [28, 29]. It should be noted that L-DNM warning system proposed in this work is entirely based on the number of clinic visits per year. That is to say, based on the data of 1 year, our model can monitor and identify the pre-outbreak signal in real time. Obviously, in order to solve practical problems, the size of small samples should also be set appropriately. That is to say, at least five samples should be given since the samples is processed by window shifting where the width of window is set to 5.

Actually, compared with traditional machine learning algorithms and deep learning approaches, L-DNM method has the following natural advantages. First, it is a model-free approach that does not require training and testing processes. There is no feature selection in L-DNM strategy, which solely depends on three statistical conditions of our model. Second, our approach can rely on small samples rather than years of time series data. So it can be applied in some developing countries that lack public health infrastructure.

## Discussion

Recently, a large outbreak of HFMD has occurred in Japan, which attracts considerable attention. According to World Health Organization (WHO), a large number of outbreaks of HFMD have been reported in countries of the Western Pacific Region over the last decade, including Japan, Malaysia and Singapore, and across China [30]. Those outbreaks not only make infected children suffer from illness, but also make parents panic. The incidence of HFMD appears to be increasing throughout the Asia-Pacific. This has prompted concerns that, without intervention, the public health impact and spread of the disease will continue to intensify. In order to combat the prevalence of HFMD, it is essential to establish a monitoring system that relies solely on robust information, such as the real-time number of clinic visits. From the successful application of the proposed approach, it is seen that the L-DNM is a model-free method, which is data-driven and thus of great potential in practical real-time monitoring.

Specifically, unlike the critical transformation analysis based on DNB of complex diseases with genomic datasets, DNB method has been improved and applied to macroscopic city networks. Using a large-scale metropolitan-wide HFMD surveillance dataset over the past decade, the landscape dynamic network marker incorporating the dynamical information of city networks is fitted to facilitate accurate and timely pre-outbreak detection. In addition, hundreds of wards can be monitored simultaneously and the outbreak risk can be assessed by landscape DNM scores, as presented in Figs. 5 and 6. It is noteworthy that L-DNM method proposed in this paper is based entirely on the number of real-time clinic visits and has obtained the remarkable results. Given more information about epidemic transmission, the L-DNM-based surveillance system is expected to reliably predict HFMD outbreaks in terms of sensitivity and accuracy.

## Conclusion

In this study, we proposed a computational method, the so-called landscape dynamic network marker (L-DNM), solely based on hospitalization records. In order to verify the effectiveness of our method, we illustrated the application of L-DNM to detect pre-outbreak signals of HFMD in Tokyo, Hokkaido and Osaka, Japan. This method can effectively identify the pre-outbreak signals with an average of 5-week window lead prior to the catastrophic transition into HFMD outbreaks. The study on the dynamical changes of clinic visits in local networks reveals the dynamic or landscapes of HFMD transmission at the network level. As the algorithm shown in Methods section, the L-DNM is easy to implement and very flexible. It is therefore of great potential in public real-time surveillance for epidemic diseases.

## Supplementary Information

**Additional file 1: Figure S1**. The landscape of L-DNM scores for 23 wards in Tokyo between the years 2009 and 2018. **Figure S2**. The city network for Hokkaido region. **Figure S3**. Forecast of seasonal HFMD outbreaks in Hokkaido between the years 2009 and 2018. **Figure S4**. The landscape of L-DNM score for 30 wards in Hokkaido between the years 2009 and 2018. **Figure S5**. The city network for Osaka, Japan. **Figure S6**: Forecast of seasonal HFMD outbreaks in Osaka between the years 2012 and 2018.

## Data Availability

The historical raw data of Tokyo region is available from Tokyo Metropolitan Infectious Disease Surveillance Center (http://survey.tokyo-eiken.go.jp/epidinfo/weeklyhc.do). The raw data of Hokkaido is available from Hokkaido Infectious Disease Surveillance Center (http://www.iph.pref.hokkaido.jp/kansen/605/data.html). The raw data of Osaka is available from Osaka Infectious Disease Surveillance Center (http://www.iph.pref.osaka.jp/infection/2-old.html).

## Abbreviations

HFMD: Hand, foot and mouth disease
L-DNM: Landscape dynamic network marker
DNB/DNM: Dynamic network biomarker/maker
SVM: Support vector machine
LSTM: Long short-term memory
AUC: Area under the curve
