# Supplementary material for: Identifying pre-outbreak signals of hand, foot and mouth disease based on landscape dynamic network marker
Source: BMC Infect Dis. 2021 Jan 15;21(Suppl 1):6. doi: 10.1186/s12879-020-05709-w (PMC7809731; doi:10.1186/s12879-020-05709-w)
Supplement: Supplementary file 1 — Additional file 1: Figure S1. The landscape of L-DNM scores for 23 wards in Tokyo between the years 2009 and 2018. Figure S2. The city network for Hokkaido region. Figure S3. Forecast of seasonal HFMD outbreaks in Hokkaido between the years 2009 and 2018. Figure S4. The landscape of L-DNM score for 30 wards in Hokkaido between the years 2009 and 2018. Figure S5. The city network for Osaka, Japan. Figure S6: Forecast of seasonal HFMD outbreaks in Osaka between the years 2012 and 2018. [file 12879_2020_5709_MOESM1_ESM.pdf]

Supplementary Information of Identifying Pre-outbreak Signals of Hand, Foot and Mouth Disease Based on Landscape Dynamic Network Marker

Xuhang Zhang, Rong Xie, Zhengrong Liu, Yucong Pan, Rui Liu and Pei Chen

Supplementary Figures

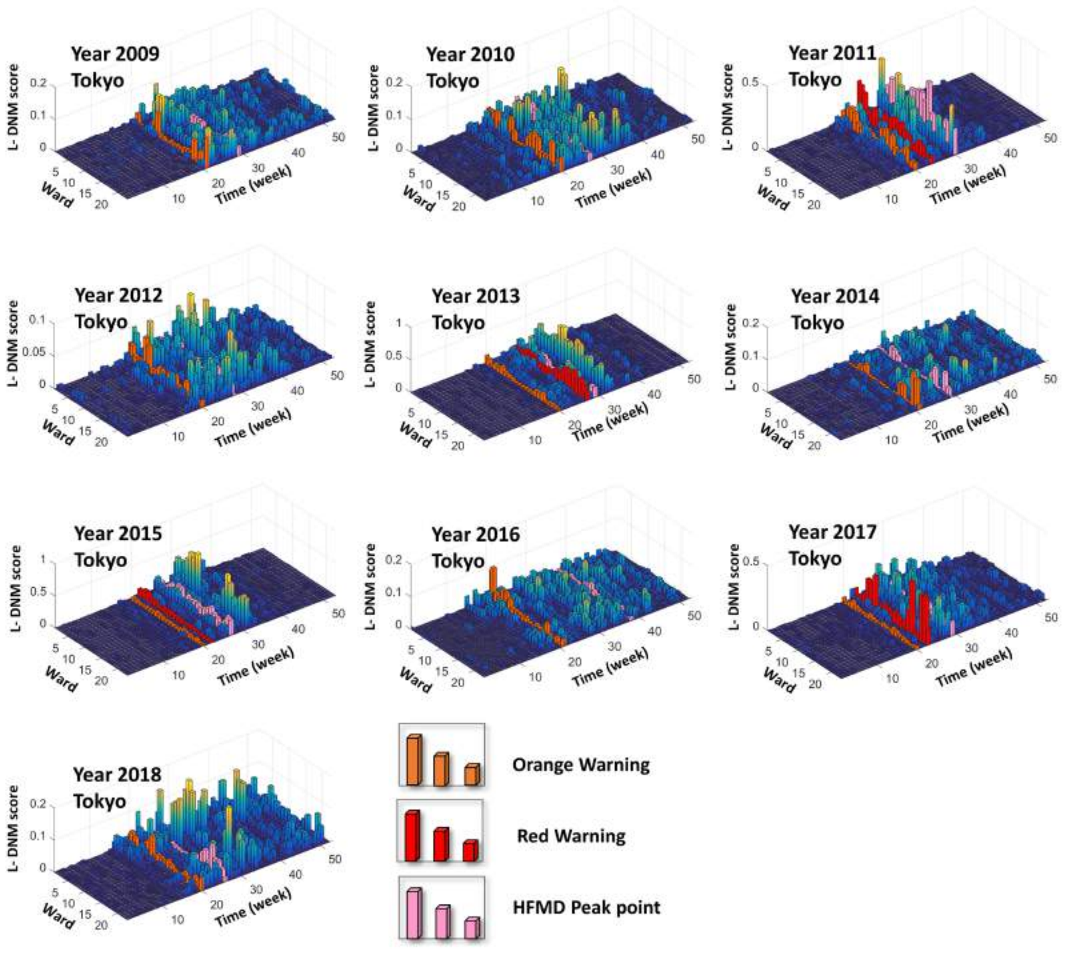

Figure S1: The landscape of L-DNM scores for 23 wards in Tokyo between the years 2009 and 2018. The orange column point to the first appearance of warning signals, the red column indicates that the scale of infection is expanding and the pink column indicates an outbreak. Obviously, the warning signals are sensitive and effective.

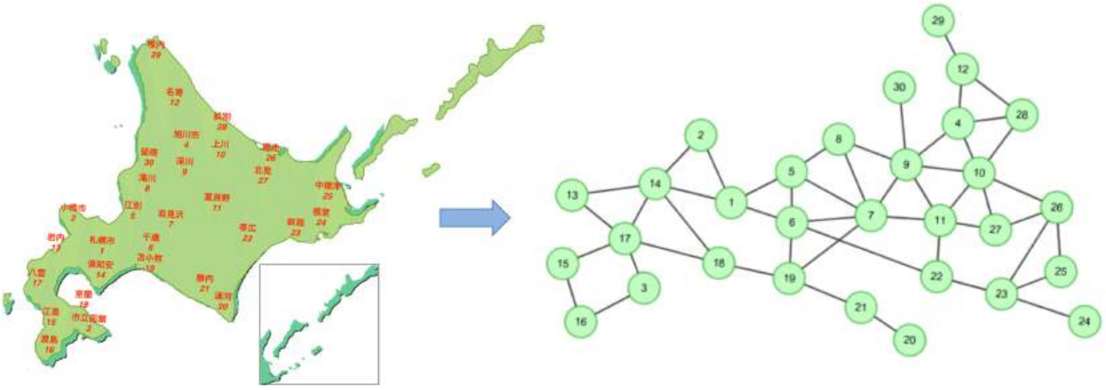

Figure S2: The city network for Hokkaido region. Based on the geographic distribution of 30 districts and their adjacent relationship, a 30-node network model is constructed for analysis.

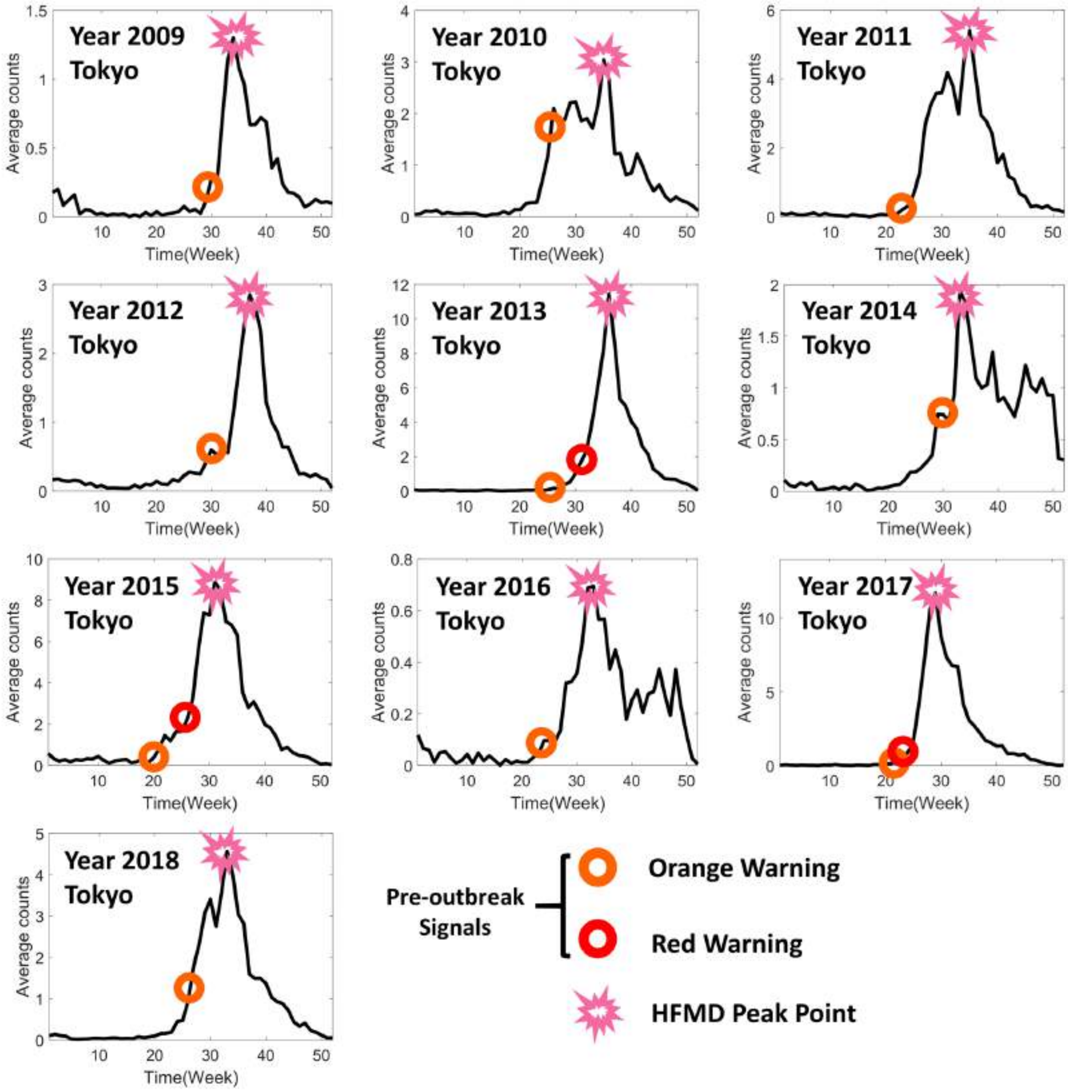

Figure S3: Forecast of seasonal HFMD outbreaks in Hokkaido between the years 2009 and 2018. In each figure, the y-axis is the average number of patients in each clinic; the x-axis represents the period from the first week of the year to the last week of the year. Besides, the markers of different colors and shapes markers are used to identify warning signals or outbreak points. Clearly, when the actual number of clinic visits has not increased significantly, significant changes in L-DNM scores have been detected, indicating the presence of pre-outbreak signals.

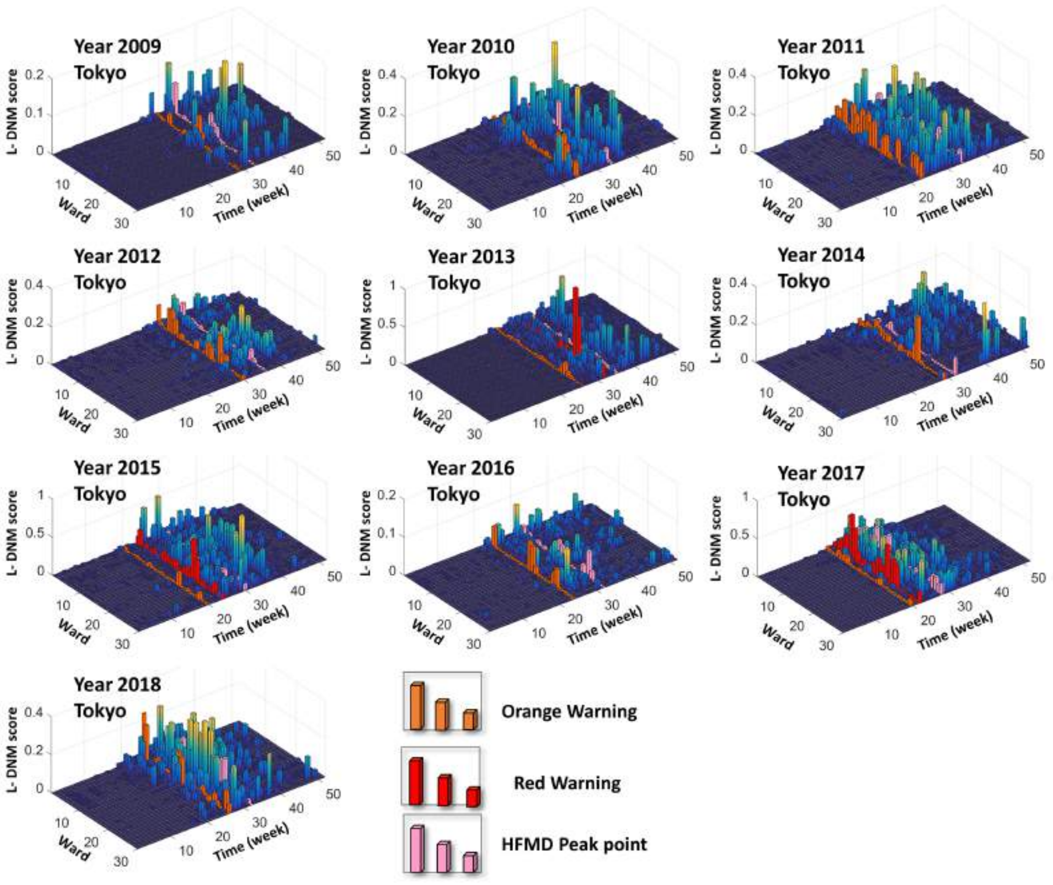

Figure S4: The landscape of L-DNM score for 30 wards in Hokkaido between the years 2009 and 2018. In each landscape figure, the L-DNM scores of 30 wards in Hokkaido are presented annually. The orange column point to the first appearance of warning signals, the red column indicates that the scale of infection is expanding and the pink column indicates an outbreak. Clearly, the warning signals are earlier than the outbreaks.

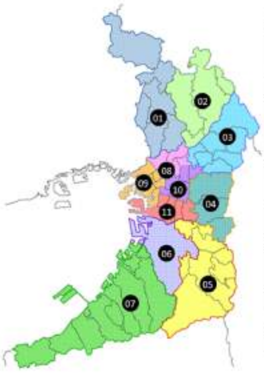

ブロック	市町村区分：所管区域	所管保健所
01 豊能	豊中市	豊中市保健所
	吹田市	吹田保健所
	池田市、箕面市、能勢町、豊能町	池田保健所
02 三島	高槻市	高槻市保健所
	茨木市、摂津市、島本町	茨木保健所
03 北河内	枚方市	枚方市保健所
	寝屋川市	寝屋川市保健所
	守口市、門真市	守口保健所
	四條畷市、大東市、交野市	四條畷保健所
04 中河内	東大阪市	東大阪市保健所
	八尾市	八尾市保健所
	柏原市	藤井寺保健所
05 南河内	藤井寺市、松原市、羽曳野市	藤井寺保健所
	富田林市、河内長野市、大阪狭山市、太子町、河南町、千早赤阪村	富田林保健所
06 堺市	堺市	堺市保健所
07 泉州	和泉市、高石市、泉大津市、忠岡町	和泉保健所
	岸和田市、貝塚市	岸和田保健所
	泉佐野市、泉南市、阪南市、田尻町、熊取町、岬町	泉佐野保健所
08 大阪市北部	北区、都島区、淀川区、東淀川区、旭区	大阪市保健所
09 大阪市西部	福島区、此花区、西区、港区、大正区、西淀川区	
10 大阪市東部	中央区、天王寺区、浪速区、東成区、生野区、城東区、鶴見区	
11 大阪市南部	阿倍野区、住吉区、住之江区、東住吉区、平野区、西成区	

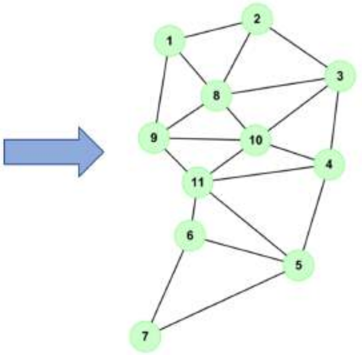

Figure S5: The network for Osaka, Japan. Based on the geographic distribution of 11 districts and their adjacent relationship, a 11-node network model is constructed for analysis.

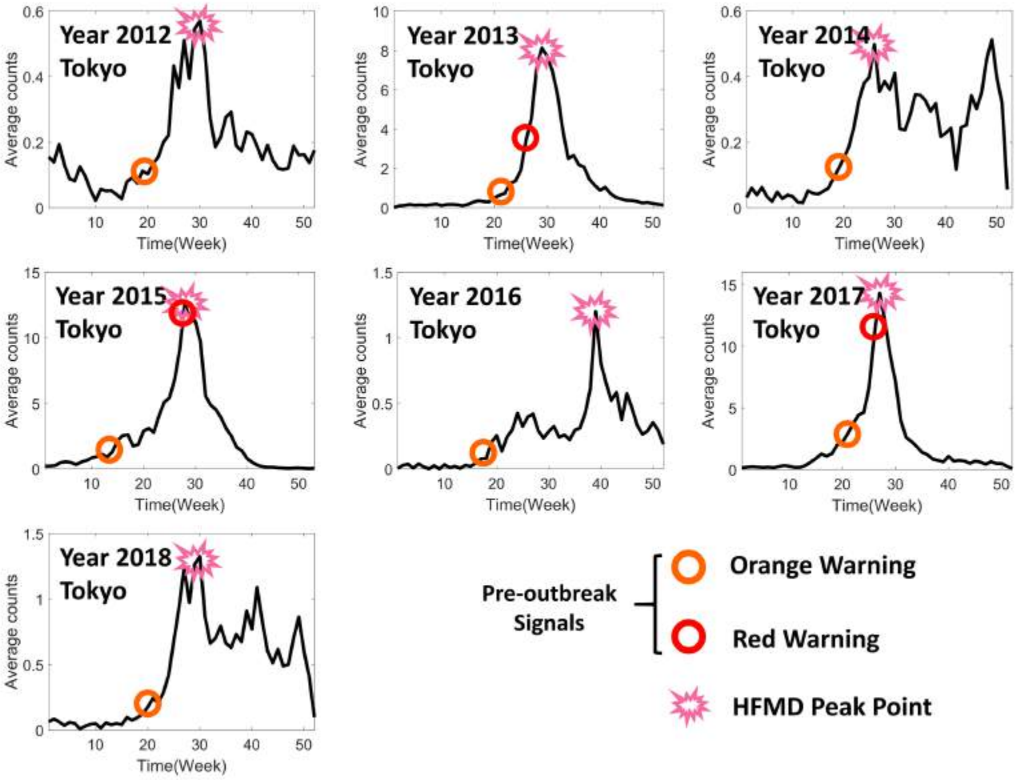

Figure S6: Forecast of seasonal HFMD outbreaks in Osaka between the years 2012 and 2018. In each figure, the y-axis is the average number of patients in each clinic; the x-axis represents the period from the first week of the year to the last week of the year. Besides, the markers of different colors and shapes are used to identify warning signals or outbreak points. Seven HFMD outbreaks occurred in Osaka from year 2012 to 2018, among which L-DNM method provided pre-outbreak signals for five outbreaks accurately.
